# Co-Inoculation of Organic Potato with Fungi and Bacteria at High Disease Severity of *Rhizoctonia solani* and *Streptomyces* spp. Increases Beneficial Effects

**DOI:** 10.3390/microorganisms9102028

**Published:** 2021-09-25

**Authors:** Orsolya Papp, Tamás Kocsis, Borbála Biró, Timea Jung, Daniel Ganszky, Éva Abod, Imre Tirczka, Franciska Tóthné Bogdányi, Dóra Drexler

**Affiliations:** 1 Hungarian Research Institute of Organic Agriculture (ÖMKi), 1033 Budapest, Hungary; daniel.ganszky@biokutatas.hu (D.G.); t.bogdanyi.franciska@gmail.com (F.T.B.); dora.drexler@biokutatas.hu (D.D.); 2 Department of Food Microbiology, Hygiene and Safety, Hungarian University of Agriculture and Life Sciences, 1118 Budapest, Hungary; kocsis.tamas.jozsef@uni-mate.hu; 3 Department of Agri-Environmental Sciences, Faculty of Horticulture, Szent István University, 1118 Budapest, Hungary; 4 Brightic Research Ltd., 2626 Nagymaros, Hungary; totimke@gmail.com; 5 Department of Horticulture, Faculty of Technical and Human Sciences, Sapientia Hungarian University of Transylvania, 540485 Târgu-Mureș, Romania; abod.eva@ms.sapientia.ro; 6 Department of Agroecology and Organic Farming, Hungarian University of Agriculture and Life Sciences, 2100 Gödöllő, Hungary; Tirczka.Imre@uni-mate.hu

**Keywords:** potato, organic, microbial inoculation, crop protection, *Pseudomonas*, *Trichoderma*, *Bacillus*, *Streptomyces*, *Rhizoctonia*

## Abstract

Rhizobacteria-based technologies may constitute a viable option for biological fertilization and crop protection. The effects of two microbial inoculants (1) PPS: *Pseudomonas protegens*, *P. jessenii* and *Stenotrophomonas maltophilia* biocontrol bacterium strains and (2) TPB: *Trichoderma atroviride*, *Pseudomonas putida*, and *Bacillus subtilis* fungi, bacteria biocontrol, and biofertilizer combinations were examined on potato (*Solanum tuberosum* L. var. Demon) in three consecutive years in irrigated organic conditions. The number of tubers showing symptoms of *Streptomyces* sp. and *Rhizoctonia* sp. was recorded. The severity of symptoms was evaluated based on the damaged tuber surface. There was a large annual variability in both the symptoms caused by soil-borne pathogens, and the effect of bio-inoculants. In the first and second year, with a stronger *Rhizoctonia* and *Streptomyces* spp. incidence, the bacterial and fungal combination of TPB inoculums with both the potential plant nutrition and biocontrol ability of the strains seemed to have a better efficiency to control the diseases. This tendency was not supported in the third year, and this may be attributed to the relatively high natural precipitation. Further studies are required to investigate the agronomic benefits of these inoculants and to tailor their application to the soil microbial characteristics and weather conditions.

## 1. Introduction

The application of beneficial microorganisms in sustainable agriculture is one of the most popular research topics today [1,2]. It is especially important to utilize biological solutions in organic agriculture where there is a high demand for alternatives of chemical pesticides against soil-borne pathogens [3]. The soil–plant–microbiome ecological system has different modes of action. The literature describes three main mechanisms: (1) increased plant nutrient supply through biological nitrogen fixation, nutrient exploration, or nutrient transport [4]; (2) induced systemic resistance against plant pathogens by competition for space and nutrients, antibiosis, parasitism, and degradation of inhibitors [5]; and (3) direct enhancement of plant growth: production of plant hormone-like substances, plant growth regulators (PGR) [6]. These projected effects can overlap and interact in many cases, and different microorganisms may also have more than one effect, which might change with plant phenology and environmental conditions [7]. Although the main mechanisms are recognized, the understanding of this complex system is far from complete, therefore new research data are needed that were obtained in real-life agronomic conditions.

Potato (*Solanum tuberosum* L.) is one of the most valuable food crops. In addition to its agricultural importance, the potato tuber is a biologically unique formation, as it is a bifunctional root involved in carbohydrate storage and vegetative propagation [8]. During crop production, many biotic and abiotic stress-factors affect the potato plants. The current practices of using agrochemicals against these stress factors can often result in severe environmental pollution problems [9]. Due to these consequences, growers are highly interested in sustainable production technologies, including organic farming practices [10]. The use of alternative and environmentally friendly solutions is crucial in replacing synthetic inputs with organic materials while improving the chemical, physical, and biological characteristics of soils [11,12].

Among beneficial microbes, the bioeffector (BE) microorganisms and solutions are frequently used in sustainable crop production systems [13,14,15]. These bioeffective organisms can reduce or replace mineral fertilizers and chemical pesticide inputs, which in turn leads to a more sustainable agricultural crop production [16]. Aside from strain selection of biofertilizers, the appropriate environmental conditions are of high importance when using a BE. The most frequently applied strains are active in plant-nutrition, i.e., the nitrogen-fixation and/or in phosphorus (P) and potassium (K)-mobilization, so as to reduce or replace inorganic fertilizers [17,18]. In Hungary, there are more than 140 different products containing microbial inoculants. These products are registered as “plant strengthening products” (PSP) or biofertilizers. Among them, only 45 are registered for application among organic conditions, mainly for administrative reasons [19]. It is not always clear whether these microbial products function solely as registered (as PSPs), or also as “plant protecting products” (PPP) or biopesticides. Unfortunately, the efficacy of these two bioeffects (the plant strengthening and/or the plant protecting effects) is not always reliable in any of the registered inoculant-based products.

It is clear, however, that the demand for special biofertilizers/biopesticides with known plant-nutritive and plant-protective potential is increasing. Pesticide use is set to be reduced by 50% during a 10-year period (up until 2030) in the EU, and it is clear that biological solutions provide realistic alternatives to the biocidal compounds of xenobiotics [3].

There are more than 60 bacterial genera isolated from the potato rhizosphere [20], but the potential and actual effects of some beneficial microorganisms are still unexplored. Furthermore, the knowledge of their colonization abilities and functioning during variable environmental conditions is still rather limited [1]. There are some detailed publications about the biocontrol activities of several bacteria isolated from the potato rhizosphere and their application against plant-pathogenic fungi [21,22]. Two of the most widely used genera are *Pseudomonas* and *Bacillus*, but the results are mainly based on in vitro or on greenhouse experiments [1,20]; therefore these successful applications need to be tested in in situ environmental conditions. Due to the annual variability of environmental stress factors, when a 50% growth promotion is induced in a greenhouse, the same BE or product may cause only 10–15% benefits in the field [23]. The main challenge is that the effects observed in a controlled environment may not manifest among field conditions as they are frequently modified by biotic and abiotic factors of the environment [13,14,24]. This is especially true for combined, second generation inoculants, where the used microorganisms are often highly adapted to the environment and to host organisms [25]. In summary, there are insufficient data regarding the application of microbial inoculants in open field experiments, and regarding the use of plant growth promoting rhizobacteria (PGPR) and their efficacy against the pathogens of potato [1].

*Streptomyces* spp. are spore-forming bacteria that are commonly found in the soil. Among the many species of the genus, only a few act as plant pathogens, causing a tuber disease known as common scab. The symptoms of scab are different skin defects, which reduce the commercial value of the crop [26]. *Streptomyces* sp. is a decomposer of organic residues; therefore it is capable of surviving indefinitely as long there is organic matter present in the soil [26,27]. Regarding the regular cattle manure usage of organic farms, infection of *Streptomyces* sp. is always expected despite the applied crop rotation practices. Therefore, research on effective plant protection methods is highly relevant for organic potato cultivation. The microbial composition of soil is a poorly understood factor in the development of scab symptoms. The soil-derived endophytic microbial community has been shown to differ among plant cultivars, fields, and tillage practices; therefore, dedicated research is necessary to study the potato rhizosphere that in turn may uncover correlations with scab susceptibility [26]. 

*Rhizoctonia solani* is a widespread fungus in all potato-growing countries [28]. It causes symptoms on the tuber, stem, and stolon of potato [27,29], and among these symptoms, the black scurf—that is actually the sclerotia of the fungus on the tuber—is the most known symptom. The pathogen overwinters as sclerotia and mycelia on infected tubers, in plant residues, or in infested soil [27]. Black scurf leads to yield loss and reduces tuber quality, and therefore marketable yield [28,29]. Currently, the complete control of *Rhizoctonia* sp. is not possible, but the severity of the disease may be limited by several crop management and protection strategies [27]. The biological control of *Rhizoctonia* sp. has been demonstrated in some cases and may provide effective and sustainable management solutions [29]. However, disease control is not always consistent [29,30]. One suggested way to improve biocontrol is to use multiple antagonists in effective combinations [29,30,31]. The benefits of this approach include multiple mechanisms of action, synergistic effects, and wider ecological ranges of activity [29,30].

During this study, the combined effects of two microbial inoculants were evaluated, containing (1) only biocontrol type of bacteria, i.e., *Pseudomonas* and *Stenotrophomonas* strains, selected previously in trials conducted in a controlled environment, and (2) strains functioning as biocontrol, biofertilizer, and P-mobilizer agents, and including both bacteria and fungi, i.e., *Trichoderma* sp. fungi (biocontrol), *Pseudomonas* sp. (PGPR), and a *Bacillus* bacterium strain (potential P-mobilizer), respectively (proven on tomato) [13,14]. Experiments were performed in an organic open field site in order to fill the knowledge gap on the potential efficacy of microbial inoculants under real-life organic farming conditions. The experiment was performed in three consecutive years to assess the annual variability and reliability of the applied microorganisms. We wanted to test whether *Rhizoctonia* infection has a synergistic correlation with the occurrence of a *Streptomyces* infection.

## 2. Materials and Methods

### 2.1. Study Site and Field Experiment

The field experiment was conducted at the Organic Educational Farm of the Hungarian University of Agriculture and Life Sciences, MATE (formerly known as Szent István University) at Babatpuszta, Hungary, (47.619160° N–19.380486° E) between 2016 and 2018. Organic farming practices have been used for more than a decade on the experimental site. The dominant soil type on the site is Haplic Luvisol. Each plot contained 48 potato plants (*Solanum tuberosum* var. Demon). The size of an experimental plot was 9.3 m^2^ with 70 × 30 cm spacing. Each treatment had four replicates and the plots were separated and surrounded by a minimum of two buffer rows in every direction.

Following the requirement of crop rotation of organic farming, the experimental site was established at different locations on the farm each year. In the first two years, the previous crop was chickpea, and fallow in the third year. No copper treatments were used on these previous crops.

Soil characteristics were recorded every year (Table 1).

The potato field was cultivated following the common practice of the farm. Tubers were planted at 10 cm soil depth in April. After emergence, 20–30 cm tall ridges were made along the rows. Weed control was done mechanically both by a cultivator and by hand. Copper was used as a fungicide, 2 times against *Phytophthora;* and *Bacillus thuringiensis* var. *tenebrionis* was used against the potato beetle (*Leptinotarsa decemlineata*) during the season as well. Tubers were harvested from the end of August.

The experimental site was irrigated. The amount of irrigation water was determined using a tensiometer. The aim was to keep soil humidity between 100 and 250 mbar/hPa, i.e., at about 60–70% of the total water holding capacity (WHC_100%_). Therefore, the amount of irrigation water depended on the actual amount of precipitation. As a result, irrigation water was 36 mm in 2016, 85 mm in 2017, and 0 mm in 2018, because 2018 was a rather rainy year.

### 2.2. Weather Data during the Study Periods

Precipitation and temperature data during the study period were recorded (Table 2). The total precipitation was 50.3 mm, 211 mm, and 305 mm in 2016, 2017 and 2018, respectively.

### 2.3. Biofertilizer Treatments and Method of Inoculation

In the three-year field experiments, the preliminary tested microbial fertilizers [32,33] were used. Two mixtures of inoculants were tested on potato and compared to the uninoculated (water only) control (Table 3). The inoculants were prepared as follows: strains were incubated for 24 h at 28 °C in a rotary shaker (140 rpm/min) up until colony forming units (CFU) reached 10^8^ mL^−1^ concentrations in a nutrient broth substrate, and then mixed them on an equal ratio to prepare the combined inoculants. Each potato tuber was given 100 mL of inoculant in the treated plots, and 100 mL of irrigation water in the control plots at planting. The liquids were poured onto tubers in the opened furrows (at seeding), and once the treatments were applied, the tubers were immediately covered with soil. The tubers were not treated with any chemicals (bactericide, fungicide). Treatments were administered once in a season each year.

The selected microorganisms were acquired from the collection of the respective universities [32]. The isolates were local, habitat-specific, and had already undergone selection in previous experiments. The selection was based on biochemical properties, biofilm formation, mobility in soil, inorganic P-mobilization, alkaline phosphatase, and alkaline protease activities. The bioeffector strains were used in a concentration of 1.33% (*v/v*), i.e., 0.2 ml per plant. PPS and TPB, as three-component biological fertilizers were both applied according to the supplier’s protocol [13,14,32].

### 2.4. Seasonal Variation of Soil Microbial Characteristics

The most probable number (MPN) of certain cultivable physiological groups of soil microorganisms, including aerobic/anaerobic bacteria, microscopic fungi, and *Pseudomonas* spp. was investigated seasonally during vegetation periods, using liquid forms of Nutrient (N) (Merck 105443–Merck Life Science Kft. Budapest, Hungary), Rosa-Bengal Chloramfenicol (RBC) (Merck, 100467–Merck Life Science Kft. Budapest, Hungary), and King-B (KB) (Biolab, KAB30500–Biolab Zrt. Budapest, Hungary) substrates [34]. Microbial numbers were calculated based on a three-fold dilution series, based on the number of positive tubes, using a three-digit code. The statistical method of [35] was applied to calculate MPN values. 

Soil samples were taken three weeks after inoculation (St-1, in April), at the 60% of flowering (St-2, in June) and at harvest (St-3, in August), and stored in 5 °C before microbiological investigations, preferably only for a maximum of 48 h.

### 2.5. Sample Examination of Potato Tubers and Data Analysis

Samples of 100 tubers were taken randomly of each plot at harvest. Tubers showing symptoms of *Streptomyces* and *Rhizoctonia* infection were counted and additionally, in 2017 and 2018, symptom severity was evaluated (surface %).

To analyze the infection rates of tubers, binomial logistic regression was used because it shows the probability of infections [36]. For the analysis of infection severity, the nonparametric Kruskal–Wallis H test was used in order to determine if there were statistically significant differences between the ranking of symptom severity variables of treatments. Correlation regression analysis was also performed to study the interrelation between the two studied disease symptoms in every year (2016, 2017, and 2018). The used statistical software was IBM SPSS 22 (IBM, Armonk, NY, USA).

## 3. Results and Discussion

### 3.1. Streptomyces sp. Infection during the Three Consecutive Test Years

#### 3.1.1. Number of *Streptomyces* sp. Infected Tubers

**Figure 1:**
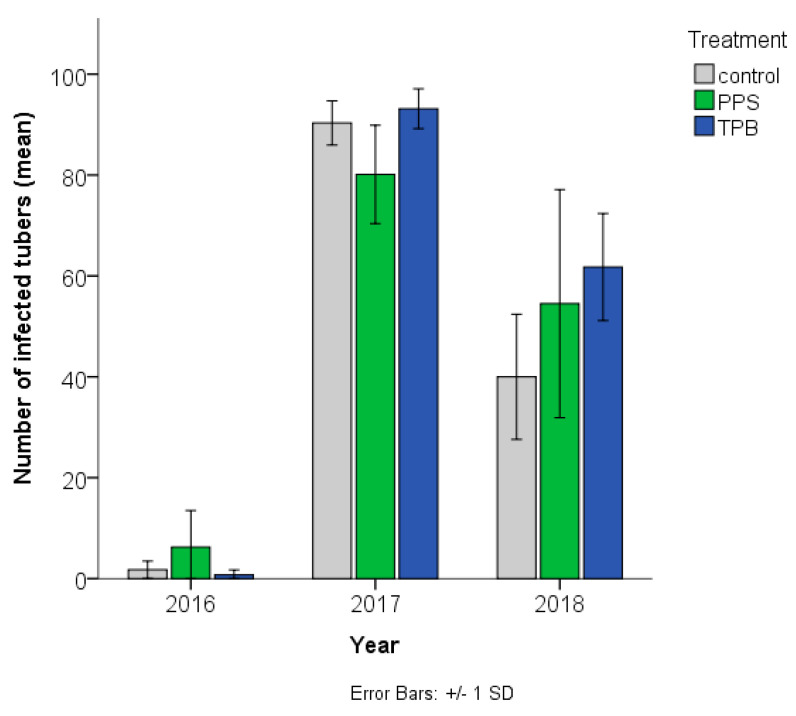
Number (means + standard deviation) of *Streptomyces* sp.-infected potato tubers in samples in the three different treatments, in three consecutive years (average of samples). Treaments: Control (Without any microbial treatments), PPS contains *Pseudomonas protegens*, *Pseudomonas jessenii*, and *Stenotrophomonas maltophilia*; TPB contains *Trichoderma atroviride*, *Pseudomonas putida*, and *Bacillus subtilis. n* = 4 in each group.

**Figure 2:**
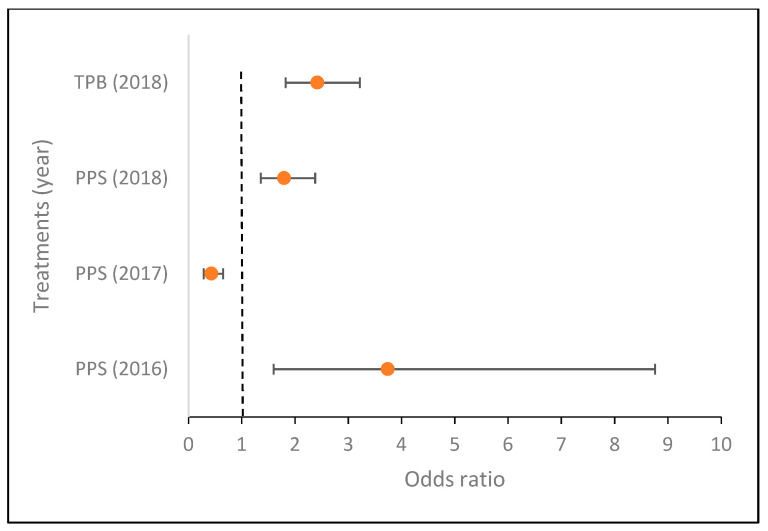
Effects of the four treatments with significant effects on the likelihood of *Streptomyces* sp. tuber infection (compared to control) assessed in three consecutive years. Treatments: PPS contains *Pseudomonas protegens*, *Pseudomonas jessenii*, and *Stenotrophomonas maltophilia*; TPB contains *Trichoderma atroviride*, *Pseudomonas putida*, and *Bacillus subtilis.* The vertical (Y) axis shows the treatments with significant results, the horizontal (X) axis shows the odds ratios, *n* = 400 in each group.

Compared to the control, the occurrence rate of *Streptomyces* sp.-infected tubers decreased in case of the combined (fungus + bacteria) PPS inoculation in 2017 but increased in 2016 and 2018; while it increased in the case of TPB inoculation in 2017 and 2018 as well (Figure 1 and Figure 2).

A binomial logistic regression was performed to assess the effects of treatments on the likelihood of *Streptomyces* sp. tuber infection each year (Figure 2). The treatment predictor variable was statistically significant in all three cases: in 2016 (χ^2^(2) = 23.518; *p* < 0.0005), the model explained 8.40% (Nagelkerke R2) of the variance in *Streptomyces* sp. infection and correctly classified 97.1% of cases. PPS had 3.743 times higher odds of exhibiting *Streptomyces* sp. infection than the control (*p* = 0.002). In 2017, (χ^2^(2) = 34.893; *p* < 0.0005) the model explained 5.5% (Nagelkerke R2) of the variance in *Streptomyces* sp. infection and correctly classified 87.8% of the cases. PPS had 0.432 times higher odds of exhibiting *Streptomyces* sp. infection than the control (*p* < 0.0005). In 2018, (χ^2^(2) = 39.567; *p* < 0.0005) the model explained 4.3% (Nagelkerke R2) of the variance in *Streptomyces* sp. infection and correctly classified 58.8% of the cases. In the treatment predictor variable, PPS had 1.797 times higher odds of exhibiting *Streptomyces* sp. infection than the control (*p* < 0.0005). Furthermore, TPB had 2.422 times higher odds of exhibiting *Streptomyces* sp. infection than the control (*p* < 0.0005) in 2018.

#### 3.1.2. *Streptomyces* sp. Infection Symptom Severity

**Figure 3:**
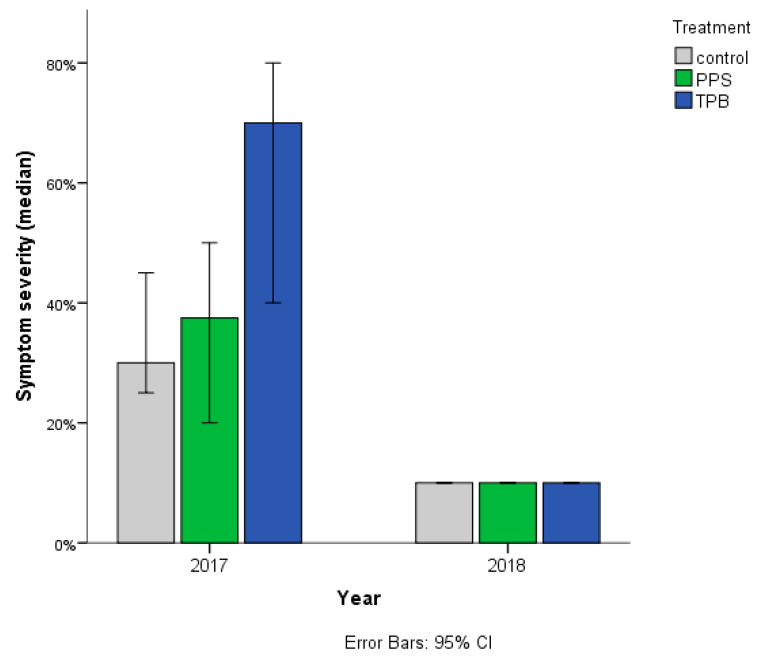
Symptom severity of *Streptomyces* sp. infection with three treatments, assessed in 2017 and 2018. Treatments: PPS contains *Pseudomonas protegens*, *Pseudomonas jessenii*, and *Stenotrophomonas maltophilia*; TPB contains *Trichoderma atroviride*, *Pseudomonas putida*, and *Bacillus subtilis*. The horizontal (X) axis shows the study years, while on the vertical (Y) axis, the symptoms in the percentage of the total yield values are shown. *n* = 40 in each group in 2017; and control *n* = 160, PPS *n* = 218 TPB, *n* = 247 in 2018.

Symptom severity of *Streptomyces* sp. infection with three different treatments is presented in Figure 3.

A Kruskal–Wallis test was conducted to determine if there were differences in the severity of symptoms of *Streptomyces* sp. infection between treatments. In 2017, there was a statistically significant difference in symptom severity among the treatments (control, PPS, and TPB—*n* = 40 groups), χ^2^(2) = 9.802, *p* = 0.007. Subsequently, pairwise comparisons were performed using Dunn’s (1964) procedure with a Bonferroni correction for multiple comparisons. Adjusted *p*-values are presented. This post hoc analysis revealed statistically significant differences in median symptom severity of *Streptomyces* sp. infection between the control (30%) and TPB (70%) (*p* = 0.019), as well as between PPS (37.5%) and TPB (70%) (*p* = 0.021) groups, but not between the control (30%) and PPS (37.5%).

### 3.2. Rhizoctonia sp. Infection during the Three Consecutive Test Years

#### 3.2.1. Number of *Rhizoctonia*-Infected Tubers

**Figure 4:**
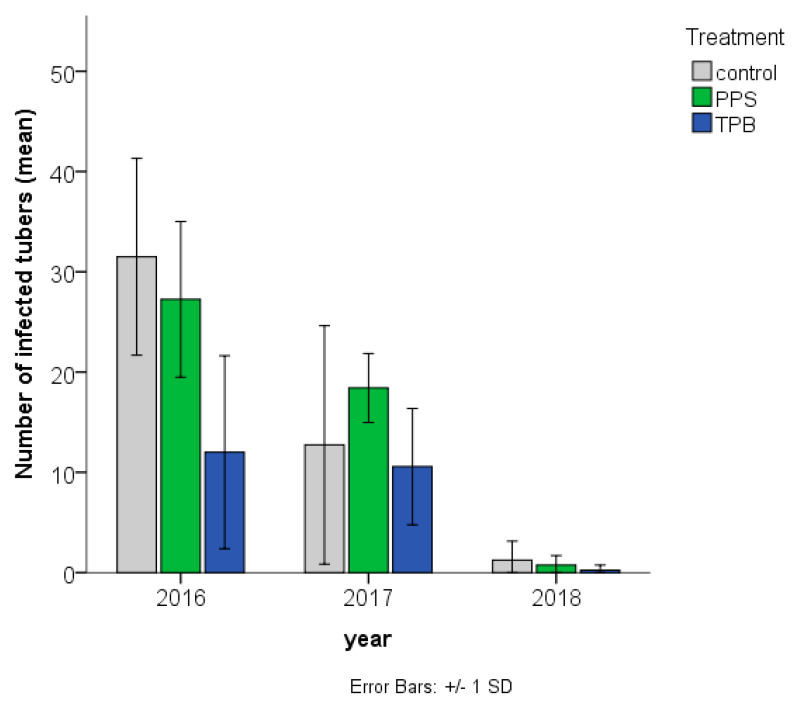
Number of *Rhizoctonia* sp. infected potato tubers in samples in the three different treatments in three consecutive years. Treatments: PPS contains *Pseudomonas protegens*, *Pseudomonas jessenii*, and *Stenotrophomonas maltophilia*; TPB contains *Trichoderma atroviride*, *Pseudomonas putida*, and *Bacillus subtilis*. The horizontal (X) axis shows the study years, while on the vertical (Y) axis, the number of symptoms (means + standard deviation) are shown in each group, *n* = 4.

Compared to the control, the number of *Rhizoctonia-*infected tubers decreased in the case of TPB inoculation in 2016, and increased in the case of PPS inoculation in 2017 (Figure 4).

**Figure 5:**
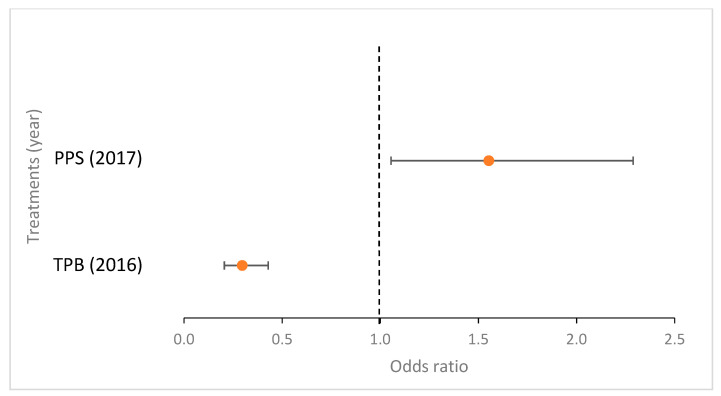
Effects of the two treatments with significant effects on the likelihood of *Rhizoctonia* sp. tuber infection (compared to control) assessed in three consecutive study years. Treatments: PPS contains *Pseudomonas protegens*, *Pseudomonas jessenii*, *Stenotrophomonas maltophilia*; TPB contains *Trichoderma atroviride*, *Pseudomonas putida*, *Bacillus subtilis*. The horizontal (X) axis shows the treatments with significant results, the vertical (Y) axis shows the odds ratios, *n* = 400 in each group.

A binomial logistic regression was performed to assess the effects of different treatments on the likelihood of *Rhizoctonia* sp. tuber infection each year (Figure 5). The treatment predictor variable was statistically significant for 2016 and 2017 results. In 2016 (χ^2^(2) = 50.392, *p* < 0.0005), the model explained 6.20% (Nagelkerke R2) of the variance in *Rhizoctonia* sp. infection and correctly classified 76.4% of the cases. In the treatment predictor variable, TPB had 0.297 times higher odds of exhibiting *Rhizoctonia* sp. infection than the control. In 2017 (χ^2^(2) = 14.022, *p* = 0.001), the model explained 2.10% (Nagelkerke R2) of the variance in *Rhizoctonia* sp. infection and correctly classified 86.4% of the cases. In the treatment predictor variable, PPS had 1.553 times higher odds of exhibiting *Rhizoctonia* sp. infection than the control (*p* = 0.026).

#### 3.2.2. *Rhizoctonia* sp. Infection Symptom Severity

**Figure 6:**
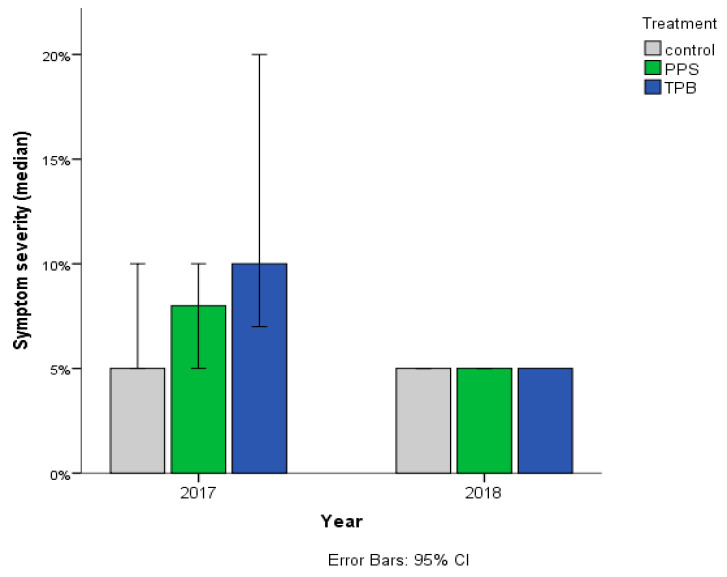
Symptom severity of *Rhizoctonia* sp. infection with three treatments, assessed in 2017 and 2018. Treatments: PPS contains *Pseudomonas protegens*, *Pseudomonas jessenii*, and *Stenotrophomonas maltophilia*; TPB contains *Trichoderma atroviride*, *Pseudomonas putida*, and *Bacillus subtilis*. The horizontal (X) axis shows the test year, the vertical (Y) axis shows the medians of symptom severity. Control *n* = 18, PPS *n* = 31, TPB *n* = 19 in 2017; and control *n* = 5, PPS *n* = 3, TPB *n* = 1 in 2018.

Symptom severity of *Rhizoctonia* sp. infection with three treatments is presented in Figure 6.

A Kruskal–Wallis test was conducted to determine if there were significant differences in the severity of symptoms of *Rhizoctonia* sp. infection among treatments. In 2017, the median of symptom severity of *Rhizoctonia* sp. infections were statistically significantly different between treatments (control—*n* = 18, PPS—*n* = 31 and TPB—*n* = 19), χ^2^(2) = 6.819, *p* = 0.033. Subsequently, pairwise comparisons were performed using Dunn’s procedure [37] with a Bonferroni correction for multiple comparisons. Adjusted *p*-values are presented. This post hoc analysis revealed statistically significant differences in median symptom severity of *Rhizoctonia* sp. infection between the control (5) and TPB (10) (*p* = 0.027), but not between the control and PPS (8) or in other combinations.

### 3.3. Seasonal Variability of Detectable Microorganisms

**Figure 7:**
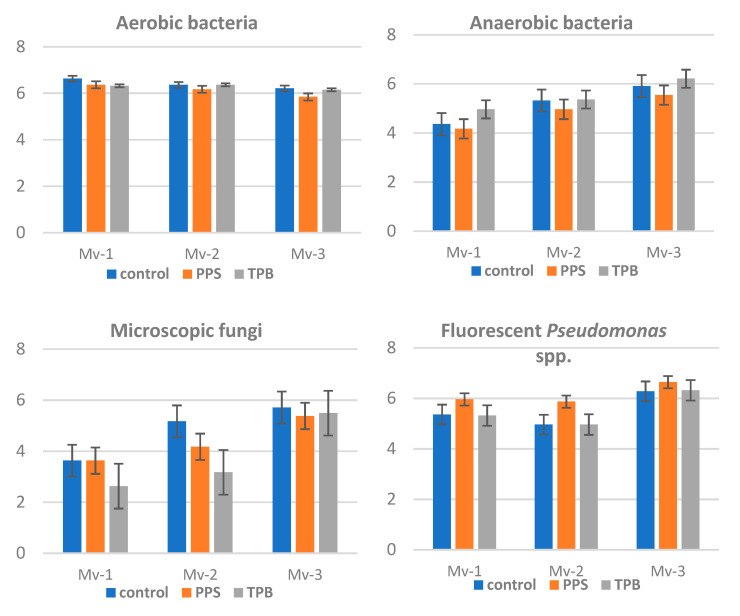
Most probable number (MPN) of certain specific microbial physiological groups, such as aerobic/anaerobic bacteria, microscopic fungi, and fluorescent *Pseudomonas* sp. count during the vegetation periods, assessed in 2016. Mv-1: sampled after 3 weeks of inoculation, Mv-2 at the 60% of flowering stage, Mv-3 at the harvest in August. Treatments: PPS contains *Pseudomonas protegens*, *Pseudomonas jessenii*, and *Stenotrophomonas maltophilia*; TPB contains *Trichoderma atroviride*, *Pseudomonas putida*, and *Bacillus subtilis.* (*n* = 3).

While the MPN values of aerobic bacteria were found to be rather constant during the vegetation period, the abundances of anaerobic bacteria and microscopic fungi were increasing, while certain fluorescein *Pseudomonads* spp. were found to be decreasing with time in the year of 2016 (Figure 7)

The tested strains of the microbial inoculants followed the general tendency of their studied microbial physiological groups. The only significant change with inoculants was found with the abundance values of *Pseudomonas* spp., where an enhanced count was recorded at the 1st sampling time (three weeks following the tuber treatment). The same tendency was found with the *Pseudomonas* spp. abundance at the third sampling time (Mv-3), in August at TPB inoculation. Those changes, however, were only tendentiously showing the effect of used microbial inoculants. No further microbial investigations recorded any significant change, therefore in 2017 and in 2018 these soil assessments were not repeated.

### 3.4. Comparison of the Effect of Microbial Inoculants on Tuber Infection

**Figure 8:**
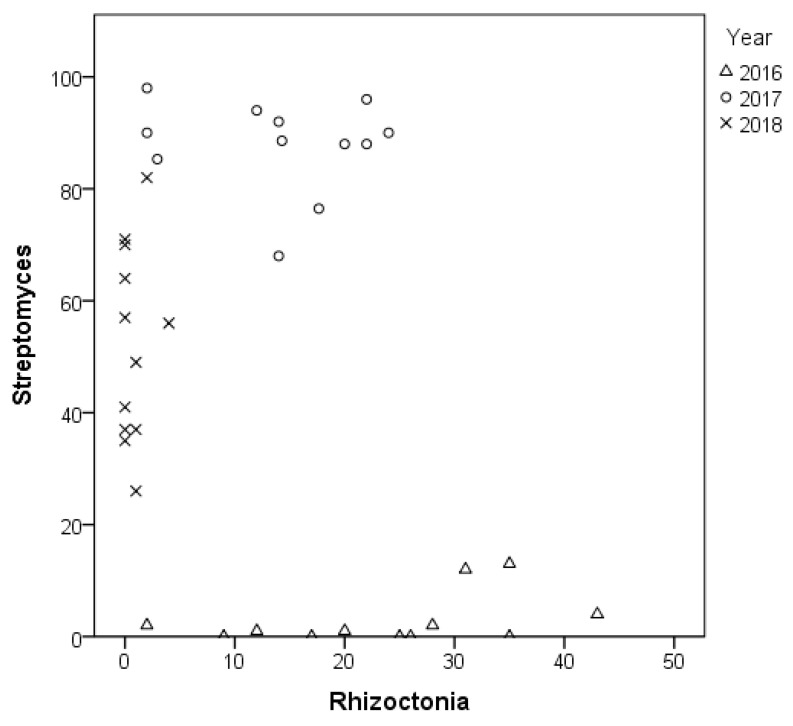
Correlation regression analysis between *Rhizoctonia* sp. and *Streptomyces* sp. infection incidence, assessed in three consecutive years (*n* = 12 in each year).

There was no statistically significant correlation (Spearman’s rho-correlation) between *Rhizoctonia* sp. and *Streptomyces* sp. infection. However, each year had a distinct pattern of the disease symptoms for both tested pathogens (Figure 8).

The two combined microbial biofertilizer inoculants tested in this study were previously tested among arable field conditions for 3 consecutive years in organic potato. Both biofertilizers/biopesticides contained 3 selected microbial strains. PPS contained biocontrol bacteria, including the fluorescent-putida type of *Pseudomonas* (*P. protegens* and *P. jessenii),* and *Stenotrophomonas maltophilia*, which was tested previously for wheat cultivation [32]. The TPB biofertilizer inoculant contained a strain of *Trichoderma atroviride*, which is a fungus frequently used in biocontrol, a biocontrol type of fluorescent-putida *Pseudomonas* (*P.* *putida*), and a spore-forming bacterium (*Bacillus subtilis*) strain, which is known for its P-mobilizing plant-nutritive effect. Based on their positive effects, both inoculants were combined by the researchers involved in the pre-selection studies. These TPB inoculants were previously used on members of the *Solanaceae* family as bioeffectors and were investigated for a 4-year of period in the Biofector project [38].

In this experiment, we wished to test the hypothesis that the specific beneficial effects of the used microbial inoculants obtained in wheat and in tomato can also be successfully repeated in potato.

The annual effects of the two tested inoculants were significantly different on *Streptomyces* sp. and *Rhizoctonia* sp. infections in every studied year (Table 4). Both PPS containing only bacteria and TPB, including the combination of bacteria and fungi had observable biocontrol ability on the disease symptoms in potato. This activity, however, proved to be inconsistent and changed by year.

The TPB treatment, which contained the *Trichoderma atroviride* fungus, the *Pseudomonas putida*, and *Bacillus subtilis* bacterial strains, microorganisms with various potential effects significantly increased the number of *Streptomyces-*infected tubers in 2018, and could decrease the number of *Rhizoctonia* sp. infected tubers in 2016 (significantly) and 2017 (a clear tendency, but no significant differences).

The PPS treatment, where the main components were the biocontrol type of bacterial strains of *Pseudomonas protegens, Pseudomonas jessenii*, and *Stenotrophomonas maltophilia* significantly decreased the number of *Streptomyces* sp.-infected tubers in 2017, but increased them in 2016 and 2018, and also significantly increased the number of *Rhizoctonia* sp. infected tubers in 2017.

No repetitively significant or unidirectional effects were observed during the three-year experiment. This fact highlights the key importance of biotic and abiotic circumstances at certain vegetation phases. 

The beneficial effects of the selected soil microbes on plant performance were demonstrated in several previous studies [39,40,41]. It is also known that the bacteria and fungi colonizing the potato rhizosphere are very specific and can be influenced by the micro-environment. The use of microbial inoculants is thus a promising approach in agriculture. The importance of this type of application is continuously increasing not only for potato, but also for several other agricultural crops including tomato [13,14], barley [23], maize [7], and alfalfa [17]. However, the proper composition of industrial biofertilizer products and the optimal methodology of inoculation including the time and application needs to be developed further. 

In the case of microbial inoculants, the most critical parameter is the survival of the strains during application within the soil–plant system. The studied three-year period provided rather fluctuating environmental circumstances. Although there was a facility for irrigation, there was still variability in the three consecutive years in water availability of soil and in the final water content. A better and more detailed understanding of the role of site-specific environmental circumstances is needed. To reach expected results, treatments may need optimization for specific soil–plant–microbe systems and for the infection characteristics of the given year.

The soils of organic farms (particularly of farms that have been under organic management for several years) have a more balanced below-ground microbial community with a well-established microenvironment [42,43,44], so it is also presumed that the rich micro-environment had a strong effect on the effectiveness of the tested bioeffectors [25].

The further exploration of inoculant use is especially important in organic farming systems, where the plant protection toolbox is limited and we need to focus on biology-based prevention solutions. 

The inoculants tested in this study contained three microbial strains each, with only bacterial and biocontrol potential in PPS and with fungal–bacterial combinations with biocontrol/biofertilizer potential in TPB. The tested combinations did not fulfil the preliminary plant protection expectations. Based on our results, different conceptual approaches may be required for developing and testing them in the future, including the investigation of applying an entire microbial consortium.

## 4. Conclusions

It is assumed that the effects of inoculations experienced in this study depended strongly on the circumstances of a given year, for example on the infection pressure by the potential pathogens, and presumably by the native microbiome of the soil. The most probable method of certain microbial physiological groups proved to be not sensitive enough to show the specific changes initiated by those microbial inoculations. Both TPB and PPS inoculants contained strains that were selected for their plant protection potential. TPB, the combination of biocontrol fungi and bacteria, was effective against *Rhizoctonia* sp. in the first two years when the severity of infection was relatively high. The same combination of TPB did not demonstrate effectiveness in the third year (with rather low infection incidences), but this was also a year with an especially heavy natural precipitation. The test results clearly demonstrated that bioeffector inoculants that were successfully developed for wheat and tomato plants did not achieve the desired plant protection effect on potatoes. This result reveals the specificity of the plant–microbial relationship. Further research is needed to study the interrelation of the tested microbial inoculant combinations in the presence or absence of soil-borne plant pathogens, and in relation to important environmental conditions. This highlights the importance of biotic and abiotic environmental factors in multifactorial soil–plant–microbial systems. 

## Figures and Tables

**Table 1:** Some soil characteristics of the experimental site in three consecutive years, at the beginning of season (sampling time: before planting, average sample of all plots).

Parameters	2016	2017	2018
pH_(H₂O)_	7.20	7.82	7.59
Dry matter content m/m%	85.3	89.3	n.d.
CaCO_3_ m/m%	2.52	1.61	5.42
Humus content m/m%	3.54	3.30	2.92
NO_3_ mg/kg	230	532	296
P_2_O_5_ mg/kg	874	1280	645
K_2_O mg/kg	376	355	334

**Table 2:** Mean monthly precipitation (mm), irrigation, and temperature (°C) at the experimental site in 2016, 2017, and 2018 (Babatpuszta, Hungary).

Years	Month	Rainfall (mm)	Temperature (°C)	Irrigation (mm)
2016	April	13.4	11.8	16
	May	12.9	15.1	-
	June	7.1	19.9	20
	July	16	19.9	-
	August	0.9	9.9	-
2017	April	55	9.1	-
	May	45	16	22
	June	37	21.1	45
	July	37	21.4	18
	August	37	n.d.	-
2018	April	14	n.d.	-
	May	51	n.d.	-
	June	131	20.4	-
	July	43.5	22.4	-
	August	65.5	23.1	-

n.d. no data.

**Table 3:** Treatments and the microorganism species content of the two inoculant mixtures used in the potato field experiment.

Treatments (Origin)	Biofertilizer Strains in Inoculants	Microorganisms in Products (CFU mL^−1^) and Application Rates (mL/Tubers)
**C** (Control)	Without any microbial treatments	100 mL water
**PPS** (Sapientia Hungarian University of Transylvania)	*P* *seudomonas protegens Pseudomonas jessenii Stenotrophomonas* *maltophilia*	Mix of strains in 100 mL suspension (CFU 1 × 10^8^ mL^−1^)
**TPB** (MATE, Hungary)	*Trichoderma atroviride* *Pseudomonas putida* *Bacillus subtilis*	Mix of strains in 100 mL suspension (CFU 1 × 10^8^)

**Table 4:** Summary of the effects of the two biofertilizer inoculant combinations on the number of *Streptomyces* sp. and *Rhizoctonia* sp. infected tubers, tested in three consecutive years.

Inoculants	Infection	2016	2017	2018
	*Rhizoctonia* sp. incidence	high	medium	weak
**TPB**	*Rhizoctonia* sp.	↓ *	↓	0
**PPS**	*Rhizoctonia* sp.	↓	↑ *	0
	*Streptomyces* sp. incidence	weak	strong	large differences
**TPB**	*Streptomyces* sp.	0	↑	↑ *
**PPS**	*Streptomyces* sp.	↑ *	↓ *	↑ *

(↑ increased, ↓ decreased, 0: no effect, *: significant difference).

## Data Availability

Data is contained within the article.
